# Latent Cytomegalovirus Infection in Female Mice Increases Breast Cancer Metastasis

**DOI:** 10.3390/cancers11040447

**Published:** 2019-03-29

**Authors:** Zelei Yang, Xiaoyun Tang, Guanmin Meng, Matthew G. K. Benesch, Martina Mackova, Ana Paula Belon, Jesus Serrano-Lomelin, Ing Swie Goping, David N. Brindley, Denise G. Hemmings

**Affiliations:** 1 Department of Biochemistry, University of Alberta, Edmonton, AB T6G 2S2, Canada; zelei@ualberta.ca (Z.Y.); xtang2@ualberta.ca (X.T.); guanmin@ualberta.ca (G.M.); benesch@ualberta.ca (M.G.K.B.); igoping@ualberta.ca (I.S.G.); 2 Cancer Research Institute of Northern Alberta, Edmonton, AB T6G 2S2, Canada; 3 Women and Children’s Health Research Institute, Edmonton, AB T6G 2S2, Canada; mackova@ualberta.ca (M.M.); ana.belon@ualberta.ca (A.P.B.); jaserran@ualberta.ca (J.S.-L.); 4 Discipline of Surgery, Faculty of Medicine, Memorial University of Newfoundland, St. John’s, NL A1C 5S7, Canada; 5 Department of Obstetrics/Gynecology, University of Alberta, Edmonton, AB T6G 2S2, Canada; 6 Department of Oncology, University of Alberta, Edmonton, AB T6G 1Z2, Canada; 7 Department of Medical Microbiology and Immunology, University of Alberta, Edmonton, AB T6G 2S2, Canada

**Keywords:** angiogenesis, collagen, glycoprotein B, interleukins, tumor growth

## Abstract

Cytomegalovirus (CMV) infects 40–70% of women, but infection has been reported in >95% of breast cancer patients. We investigated the consequences of these observations by infecting mice with mCMV or a negative control medium for 4 days, 11 days or 10 weeks to establish active, intermediate or latent infections, respectively. Syngeneic 4T1 or E0771 breast cancer cells were then injected into a mammary fat pad of BALB/c or C57BL/6 mice, respectively. Infection did not affect tumor growth in these conditions, but latently infected BALB/c mice developed more lung metastases. The latent mCMV infection of MMTV-PyVT mice, which develop spontaneous breast tumors, also did not affect the number or sizes of breast tumors. However, there were more tumors that were multilobed with greater blood content, which had enhanced vasculature and decreased collagen content. Most significantly, mCMV infection also increased the number and size of lung metastases, which showed a higher cell proliferation. Viral DNA was detected in breast tumors and lung nodules although viral mRNA was not. These novel results have important clinical implications since an increased metastasis is prognostic of decreased survival. This work provides evidence that treating or preventing HCMV infections may increase the life expectancy of breast cancer patients by decreasing metastasis.

## 1. Introduction

Breast cancer is the most common malignancy among women, and our inability to treat metastasis is a major cause of mortality. The net 5-year survival rates are 87% for all patients diagnosed with breast cancer, but once the tumors metastasize, the survival rate decreases dramatically to 22% [1]. It is, therefore, vital to develop new strategies for preventing metastasis and for treating metastatic cancer to decrease mortality. 

This study concentrates on the role of cytomegalovirus (CMV) infection on breast tumor growth and metastasis. Human CMV (HCMV) is a β-herpesvirus of the Herpesviridae family, and it produces lifelong infections [2]. Forty to 70% of the adult population is infected by HCMV in developed countries, and this rate increases to approx. 85–90% when people reach 75–80 years of age [3,4]. In fact, it has been hypothesized that the response to infection with this virus depends on the age at initial exposure, with a late exposure causing an increased risk of breast cancer [5].

HCMV infection normally causes no noticeable symptoms in immunocompetent individuals [6]. After entry into susceptible cells, HCMV produces viral proteins in three stages: immediate early (IE), early and late [7]. At each stage, these viral proteins have essential roles in establishing a productive HCMV infection. IE proteins are important for transcription regulation, early proteins are important for viral replication and late proteins are important for the assembly of new viral particles [8]. A strong innate immune response is produced during the initial HCMV infection. This is followed by an adaptive response, and both are important in the host’s defense against HCMV [9]. Once the initial infection is resolved, CMV enters a latent state where the viral genome is retained in the host with a restricted expression of viral genes [10]. In contrast to the initial active infection where virus replication is high, replication in chronic and presumed latent infections is at low subclinical levels, if present at all [11]. In fact, this latent state has been described as symbiotic since it can confer immune benefits to the host against other infections [12,13].

A reactivation to full virus replication can occur during inflammation and stress in the aging population and in immunocompromised hosts including organ transplant recipients undergoing immunosuppressive therapies [14,15,16,17]. This reactivation of HCMV infection can be life-threatening, especially in immunocompromised or critically ill patients [18].

Increasing evidence shows that HCMV infection could play a role in breast cancer [19,20]. It has been reported that 95% of women with breast cancer are HCMV-infected with a detection of viral DNA and/or proteins in breast tissues, whereas only approx. 70% of non-neoplastic women are infected [21]. Several groups support a high detection of HCMV infection in women with breast cancer and the evidence of HCMV being present in breast tumors [22,23]. HCMV proteins and DNA are abundantly expressed in sentinel lymph nodes [24] and in brain tissue [25] from breast cancer patients, suggesting that HCMV might be associated with invasiveness and metastasis. HCMV positivity is also related to a poor overall survival and a low, relapse-free survival in breast cancer patients [26]. 

We conducted preclinical studies to interpret these findings and to understand the consequences of mouse CMV (mCMV) infection on the progression of breast cancer. We used three different mouse models of breast cancer to provide the first direct evidence that mCMV infection had only a minimal effect on breast tumor growth. However, mice with a presumed latent infection had increased numbers and sizes of lung metastases and enhanced cancer cell proliferation in the metastatic nodules. Latently infected MMTV-PyVT mice also developed breast tumors with enhanced angiogenesis and decreased collagen content, both of which could promote metastasis. Although mCMV DNA was detected in breast tumors and lung metastatic nodules from the latently infected mice, no active transcription of viral genes was detected in any of the tissues examined. These results from mouse models indicate that latent mCMV infection increases the metastasis of breast cancer cells to the lungs. This raises the possibility that HCMV infection in breast cancer patients could play a crucial role in increasing mortality from metastasis. If so, then preventing or controlling the negative consequences of an HCMV infection could provide a major improvement in breast cancer treatment and patient survival. 

## 2. Results

### 2.1. mCMV Infection Had Minimal Effect on Breast Tumor Growth in Mice

We first demonstrated that an injection of the virus into mice from each mouse model produced an active infection by measuring the activity of β-galactosidase, a product of the LacZ gene, which was inserted into the IE-2 region of the mCMV genome. This modified virus has the same properties as native mCMV, but the production of β-galactosidase allows an active infection to be visualized by the conversion of its substrate, X-gal, into a blue product when the virus is actively replicating [27]. As an example, positive staining was observed in kidneys collected from C57BL/6 mice infected with active mCMV for 4 days but not in kidneys from mice injected with medium from which the virus was removed by filtration (FCMV; Figure S1a,b), UV-inactivated virus or PBS [28]. In addition, transcripts for mCMV IE-1/3 and a late viral product, glycoprotein B (gB), were both detected in the salivary gland and kidney of mice actively infected for 4 days but not in control-treated mice [28]. The number of infected cells in kidneys as detected by β-galactosidase staining did not differ among models during active infection [28].

We then tested the effects of active, intermediate and latent mCMV infections on breast tumor growth. This was achieved by waiting 4 days, 11 days or 10 weeks, respectively, after treatment with mCMV or the control solutions before inoculating syngeneic 4T1 breast cancer cells into a mammary fat pad of BALB/c mice or E0771 breast cancer cells into C57BL/6 mice. The volumes of the breast tumors in mCMV-infected BALB/c mice were not significantly different throughout tumor development compared to the control-treated mice regardless of the timing of infection (Figure S2a,c,e). In the C57BL/6 mice, there were no differences in the tumor volume in those with an intermediate or latent infection; however, mice with an active infection prior to the injection of breast cancer cells had a reduced overall estimate of tumor volume on days 15 and 17, significant in post hoc testing (Figure S2b,d,f). The tumor volume is difficult to measure accurately, especially with fluid-filled tumors, and tumor mass measurements are normally more accurate. 

**Figure 1:**
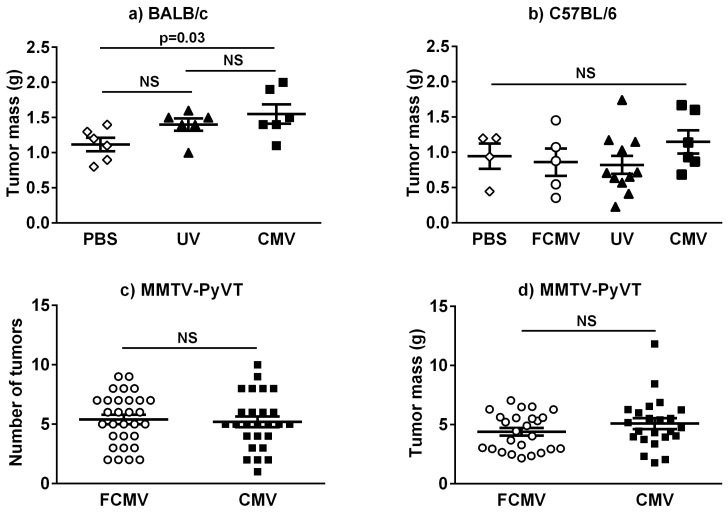
The latent mCMV infection has minimal effects on breast tumor mass. The breast tumor mass was measured at the endpoint in the latent infection models of BALB/c (**a**) and C57BL/6 (**b**) mice, n = 6 and n = 4–11, respectively. The control mice were injected with PBS, UV-inactivated CMV (UV) or a solution where active CMV was filtered out (FCMV). The total number (**c**) and total mass (**d**) of all breast tumors developed in MMTV-PyVT mice were quantified for 25 mice treated with a filtered control solution (FCMV) and for 23 mCMV-infected mice. The *p*-values were calculated by a one-way ANOVA (Figure 1a,b) or Student’s *t*-test (Figure 1c,d) with *p* < 0.05 considered to be significant. NS = not significant.

With one exception, the masses of the breast tumors at the endpoint in the BALB/c or C57BL/6 models were not significantly different in the active or intermediate treatment groups between mCMV-infected or control-treated mice from either strain (Figure S3a–d) or in the latently infected mice (Figure 1). The exception was that latently infected BALB/c mice had an increased tumor mass compared to the PBS-treated group, but there was no significant difference compared to the control treatment where mCMV was inactivated by UV exposure (Figure 1a). 

We then studied MMTV-PyVT mice, which are on a C57BL/6 background, to provide a spontaneous model of breast cancer that better resembles the human situation. mCMV or FCMV was injected at 5 weeks of age to allow at least 10 weeks to develop a latent mCMV infection before the spontaneous tumors became palpable. The number of tumors per individual mouse was highly variable, as expected in this model, with a range from 1 to 10 tumors (Figure 1c). The total burden of tumor mass per mouse (Figure 1d) and the average volume, mass or density of tumors (Table 1) did not differ between the FCMV and mCMV groups at the experimental endpoint. The time required for mice to reach the experimental endpoint was also not significantly different [28].

### 2.2. Latent mCMV Infection of MMTV-PyVT Mice Changed Breast Tumor Phenotype

**Figure 2:**
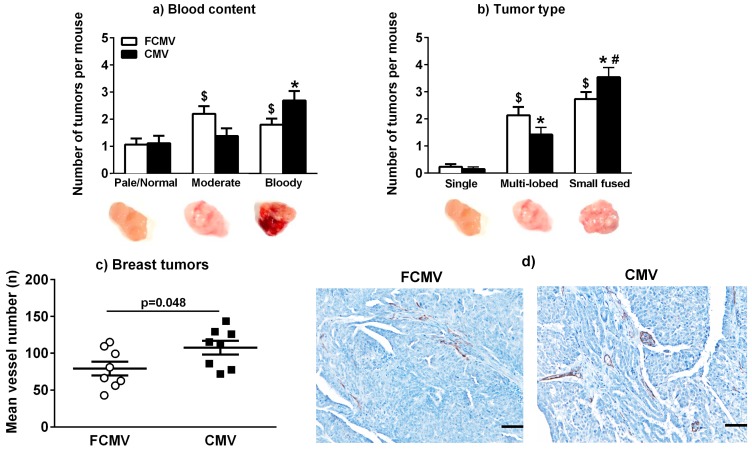
MMTV-PyVT mice latently infected with mCMV developed more breast tumors with severe phenotypes associated with metastasis. The tumors were categorized as pale/normal, moderate or bloody (**a**) or as a single tumor, multilobed or fused small tumors (**b**) in 25 control mice treated with a filtered control solution (FCMV) or in 23 mCMV-infected mice. The mean number and SEM for each type of tumor per mouse are shown, and the *p*-values were calculated by a two-way ANOVA and Tukey’s post hoc test. A significant interaction between the treatment and phenotype was found in Figure 2a (*p* = 0.009) and Figure 2b (*p* = 0.01). $ = *p* < 0.05 compared to the pale/normal or single tumor in the FCMV group; * = *p* < 0.05 compared to the pale/normal or single tumor in the mCMV group; # = *p* < 0.05 compared to multilobed in the CMV group. The number of blood vessels was assessed by the CD31 staining of sections of breast tumors from the mCMV-infected mice (**c**), n = 8. The representative images of stained sections (**d**): The images were taken with a 20× magnification lens. Scale = 100 μm. The *p*-value was calculated using a Student’s *t*-test.

Each breast tumor in the MMTV-PyVT mice was examined for different phenotypes, which included the blood content categorized as pale, moderate or bloody tumors (Figure 2a); the tumor type characterized as single tumors, multilobed or fused small tumors (Figure 2b); and other morphologies (Table S1). Although an mCMV infection did not affect the number of tumors per mouse (Figure 1c), it did change the tumor phenotypes. On average, each mouse had one tumor that had a pale appearance. In the FCMV control mice, equal numbers of tumors per mouse were characterized as moderate or very bloody. By contrast, more tumors in each mCMV-infected mouse were very bloody (dark red) as opposed to containing a moderate amount of blood (pink) (Figure 2a). Very few or no tumors per mouse were characterized as a single tumor. The numbers of multilobed and fused small tumors in each FCMV control mouse were not significantly different. By contrast, a significantly greater proportion of fused small tumors were found in each mCMV-infected mouse compared to multilobed tumors (Figure 2b). When analyzing all tumors, we determined that tumors with more severe phenotypes such as high blood content (*p* = 0.005) or fused small tumors (*p* = 0.006) were more often found to be from the mCMV-infected mice. This was not the case with tumors that were solid, soft, necrotic, disintegrated or fluid-filled (*p* = 0.303) (Table S1). In addition, the odds ratio of having a fused small tumor combined with high blood content was 2.22 times higher (95% CI 1.29, 3.84; *p* = 0.004) in the mCMV-infected group compared to the FCMV controls [28].

The observed increase in the proportion of very bloody breast tumors in the mCMV-infected MMTV-PyVT mice was further investigated by assessing the number of blood vessels by staining for the endothelial marker, CD31. Eight mice from each group were chosen randomly, and the largest tumor at the endpoint from each mouse was used for analysis. These large tumors were bloodier with more fused lobes, regardless of treatment. The average number of blood vessels observed in the stained sections of the breast tumors from the mCMV-infected mice was significantly increased compared to those from the FCMV control mice (Figure 2c,d). 

**Figure 3:**
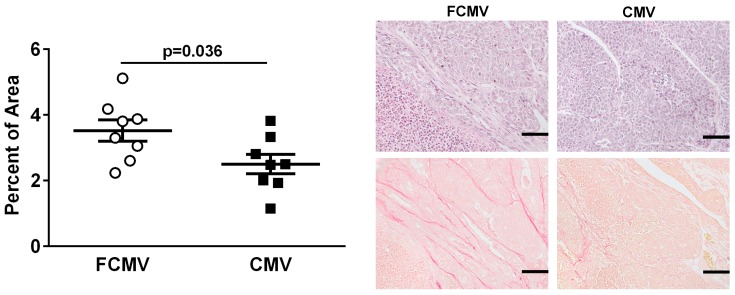
The tumors from MMTV-PyVT mice latently infected with mCMV had a lower collagen content. Collagen was quantified as the percent of total area in sections from the largest breast tumors from n = 8 mice in each group. Representative matched sections taken from the same area of the breast tumors were stained with hematoxylin and eosin for the detection of nuclei (top) and for collagen using picro-sirius red (bottom). The images were taken with a 20× magnification lens. Scale = 100 μm. The *p*-values were calculated by Student’s *t*-test.

The amount of collagen was significantly lower in the breast tumors of mCMV-infected MMTV-PyVT mice compared to the controls (Figure 3). This could have reflected an increased collagen degradation, but there were no differences in the expression of MMP-2, MMP-3, MMP-8 (a collagenase), pro-MMP9 and MMP-12 in the tumors [28].

These combined results establish that, although the mCMV infection of MMTV-PyVT mice did not affect the tumor load, there was a difference in the tumor phenotype that could modify metastasis.

We also characterized the tumors from the C57BL/6 and BALB/c mice that were injected orthotopically with E0771 or 4T1 breast cancer cells, respectively. In these cases, there were no obvious differences in the tumor phenotypes in latently infected mice of either strain. The breast tumors from BALB/c mice that were actively infected prior to the injection of breast cancer cells were also stained for CD31 and for collagen content. There were no significant differences between the UV-inactivated control and the mCMV-infected mice [28].

### 2.3. Lung Metastasis Was Increased in mCMV-Infected BALB/c and MMTV-PyVT Mice 

**Figure 4:**
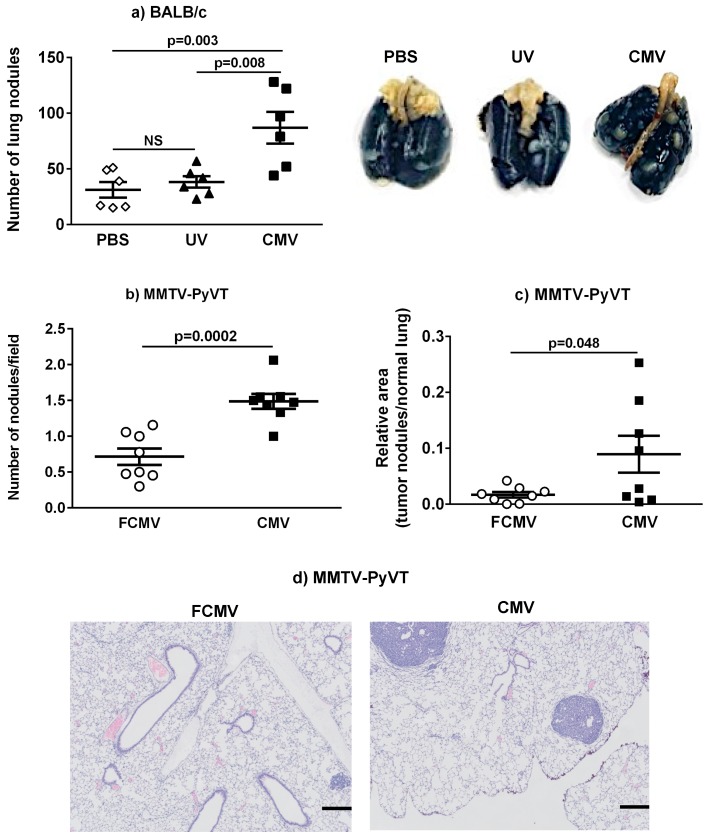
Lung metastasis was increased in mice latently infected with mCMV. The number of large lung nodules in the BALB/c model (n = 6) were quantified, and the representative lungs were depicted (**a**). Lung micro-metastasis in the MMTV-PyVT model (n = 8) was assessed by staining with hematoxylin and eosin and analyzed as the number of nodules per microscopic field at 5× magnification (**b**) and the area of nodules per lung area (**c**). The representative stained lung sections from the mice treated with a filtered control solution (FCMV) or mCMV with a 5× magnification lens are shown (**d**). Scale = 280 μm. The *p*-values were calculated by a one-way ANOVA (Figure 4a) or Student’s *t*-test (Figure 4b,c). NS = not significant.

Latently infected BALB/c mice showed a 3-fold increase in the number of metastatic nodules on the lung surface when compared to the PBS and UV-inactivated control groups (Figure 4a). However, this increase in lung metastasis was not observed in BALB/c mice with an active or intermediate mCMV infection prior to the introduction of the cancer cells (Figure S3e,f). The injection of E0771 cells into the mammary fat pads of C57BL/6 mice did not produce obvious macro- or micro-metastasis in the lungs or liver at the experimental endpoint for tumor growth [28].

Macro-metastatic nodules were not observed on lungs from the MMTV-PyVT mice, but sectioned lungs stained positively for micro-metastasis. The latent infection of MMTV-PyVT mice with mCMV resulted in 2-fold more lung nodules compared to the control mice. These nodules were also larger in the mCMV-infected group, and they occupied a 6-fold greater area of the lungs (Figure 4b–d). No significant micro-metastases were detected in the liver [28].

**Figure 5:**
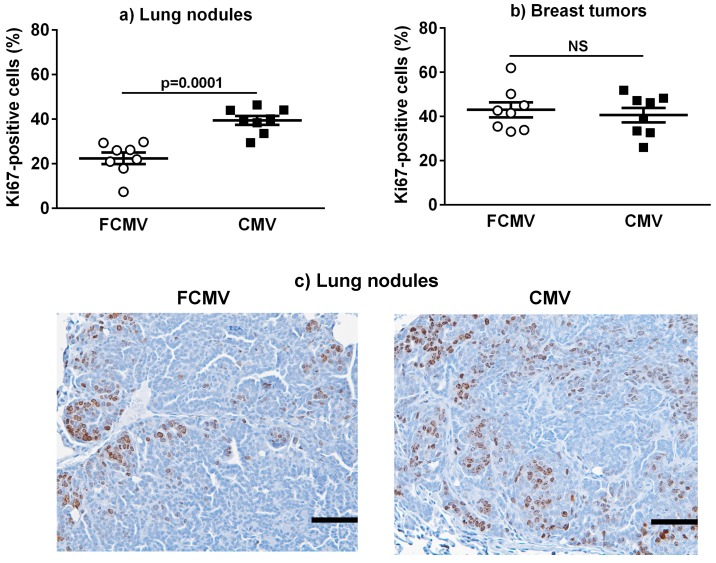
Cancer cell proliferation was enhanced in lung metastatic nodules but not in the breast tumors from MMTV-PyVT mice latently infected with mCMV. Lung metastatic nodules (**a**) and breast tumors (**b**) from mice treated with a filtered control (FCMV) or mCMV (n = 8) were stained for Ki67 to assess cell proliferation. The representative stained sections of lung nodules from FCMV- and mCMV-treated mice are illustrated (**c**). The image was taken with a 20× magnification lens. Scale = 100 μm. The *p*-values were calculated by Student’s *t*-test. NS = not significant.

Cell proliferation was determined in the lung metastatic nodules and breast tumors for the MMTV-PyVT and BALB/c mice from the active and latent models by staining for Ki67. There was a 2-fold increase in the percentage of Ki67-positive cells in the metastatic nodules of MMTV-PyVT mice that were latently infected with mCMV compared to the FCMV control mice (Figure 5a,c). A significant increase in Ki67 positivity in lung nodules was also observed in the latently infected BALB/c mice compared to both the PBS and UV controls, but this difference was not observed in the actively infected model (Figure S4a,c). By contrast, there was no significant difference in the percentage of Ki67-positive cells in breast tumors between the controls and active or latently infected mice in all models tested (Figure 5b and Figure S4b,d).

### 2.4. mCMV DNA but Not mRNA Was Detectable in Tissues, Metastatic Nodules and Tumors from Mice Latently Infected with mCMV

**Figure 6:**
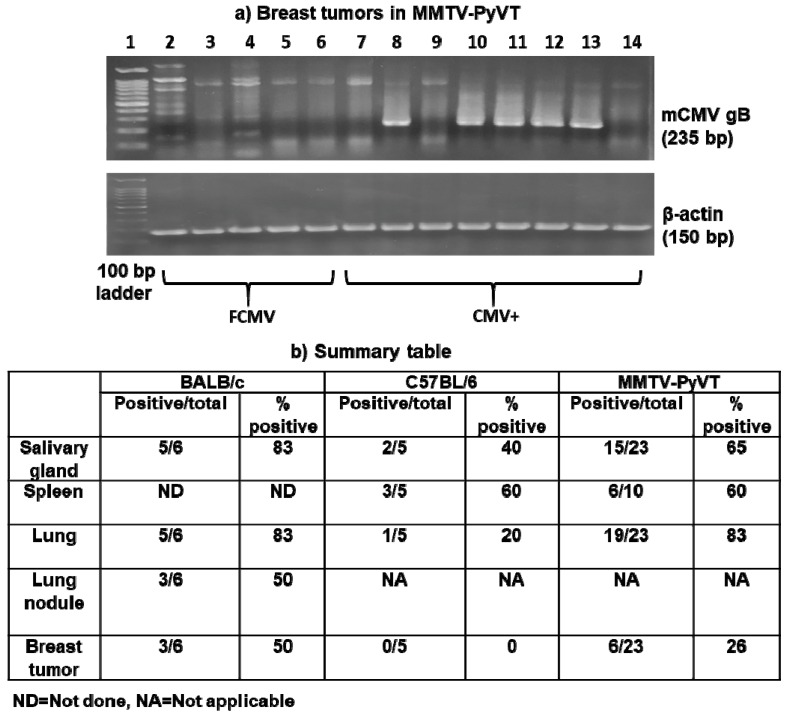
The DNA for mCMV glycoprotein B (gB) was detected in MMTV-PyVT mice latently infected with mCMV. The representative gel shows the detection of DNA for mCMV gB in breast tumors from five filtered control (FCMV; lanes 2–6) and eight mCMV-treated (lanes 7–14) MMTV-PyVT mice (**a**). Lane 1 = 100 bp ladder. A positive detection of mCMV gB DNA was identified by a band at 235 base pairs (bp). Beta-actin (150 bp) was used as an internal control. The number of tissues, nodules or breast tumors positive for mCMV gB DNA from each animal model was summarized in Panel (**b**).

We then tested for the presence of the viral genome by measuring mCMV gB DNA and for gene expression by measuring mRNA for gB, a viral gene which is transcribed at the late phase in viral replication. Salivary glands, spleens, lungs, lung metastatic nodules and breast tumors from mice latently infected with mCMV were examined. A sample was considered positive for viral gB DNA with the detection of a 235 bp band after a nested PCR amplification of the gene with β-actin as the internal control. A representative gel of the breast tumor samples from MMTV-PyVT mice is shown in Figure 6a. 

The specific detection of viral gB DNA in the tissues, metastatic nodules or tumors tested for each mouse are delineated in Table S2. Apart from 2/6 animals in the C57BL/6 model and 1/23 in the MMTV-PyVT model, there was positive detection of DNA for mCMV gB in at least one type of tissue, nodule or tumor examined from each animal (Figure 6b). BALB/c mice had macro-nodules on the surface of the lungs that were separated from the underlying lung tissue. Half of these lung nodules from the BALB/c mice were positive for viral gB DNA compared to the 5/6 positive underlying lung tissues. Macro-nodules were not present on the lungs of the C57BL/6 or MMTV-PyVT mice. Viral DNA for gB was detected in lung tissues from 83% of MMTV-PyVT mice but only 20% of the C57BL/6 mice. Viral DNA was detected in 50% and 26% of breast tumors from the MMTV-PyVT mice but none from the C57BL/6 mice. Similar results were obtained when detecting DNA for the mCMV IE-1 gene in the MMTV-PyVT model [28]. The detection of viral DNA was not observed in any control mice, including PBS, UV-inactivated or FCMV-treated mice, which indicates that the mice were seronegative at the start of the experiments [28].

mCMV gB mRNA was also measured by RT-PCR to assess if there was an active infection. However, mRNA for gB was not detected in any tissues tested including salivary glands, spleens, lungs, metastatic nodules or tumors from the three mouse models [28]. Negative results were also obtained for mRNA detection of mCMV IE-1/3 [28]. In addition, tumors and lungs were stained for β-galactosidase activity, but no detection was observed in any of the experimental models [28]. Similar results were observed for the active infection models at the tumor endpoint [28].

### 2.5. Cytokine Expression Was Altered in Breast Tumors and Plasma from Mice With Latent mCMV Infections

**Figure 7:**
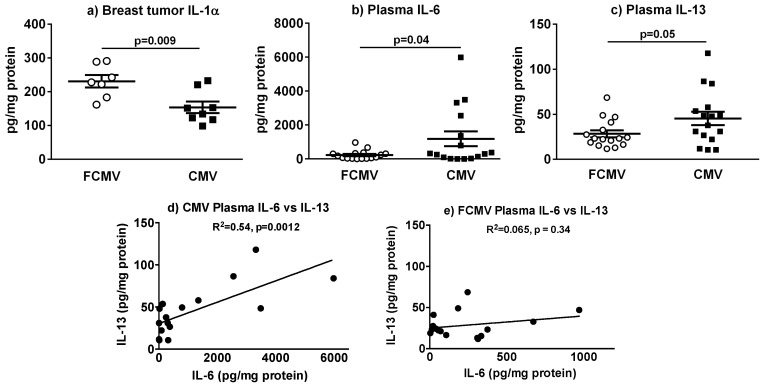
Cytokine concentrations differed in breast tumors and plasma from MMTV-PyVT mice latently infected with mCMV. Breast tumor levels of IL-1α, n = 8 (**a**). Plasma concentrations of IL-6 (**b**) and IL-13 (**c**), n = 16. The regression analysis of plasma concentrations IL-6 versus IL-13 in mCMV-infected (**d**) and mice treated with a filtered control solution (FCMV) (**e**). The *p*-values were calculated by Student’s *t*-test.

We analyzed the plasma and tumors for 32 cytokines/chemokines using a Discovery 32-plex Array and only found the following significant differences: Breast tumors from MMTV-PyVT mice latently infected with mCMV had a decreased expression of interleukin-1-alpha (IL-1α) (Figure 7a). mCMV-infected mice had increased plasma concentrations of interleukin-6 (IL-6) and interleukin-13 (IL-13) compared to FCMV-treated mice (Figure 7b,c). There was a positive correlation between these concentrations of IL-6 and IL-13 for the mCMV-infected mice (Figure 7d; *p* = 0.0012) but not for the FCMV-treated mice (Figure 7e; *p* = 0.34).

## 3. Discussion

The association between HCMV and breast cancer has been studied over the past two decades, but the role of HCMV still remains controversial [29]. Very little is known about the mechanisms by which CMV infection could modify tumor progression. We investigated the effects of mCMV infection on breast cancer progression using three mouse models of breast cancer and showed, for the first time, that latent mCMV infections prior to tumor development increase metastasis to the lungs. 

We detected viral DNA in the latently infected mice at least once when we analyzed the metastatic nodules, breast tumors or various tissues including salivary gland, spleen or lungs. We determined if the presence of a breast tumor or metastasis initiated mCMV reactivation. This state of a latent CMV infection resembles that commonly found in immunocompetent people [6]. CMV latency is established in hematopoietic progenitor cells and in cells of the myeloid/monocyte lineage that serve as a reservoir for the viral genome [30,31]. The reactivation of CMV occurs in differentiated myeloid macrophages and dendritic cells [32,33,34]. These immune cells infiltrate the tumor site, and they are important components of the tumor microenvironment [35]. The role of these immune cells in our animal models is unknown. The presence of DNA but the lack of detection of mRNA for mCMV gB, IE-1/3 and β-galactosidase activity in tissues, nodules and tumors confirmed latency. This surprised us since we had predicted that tumor-induced inflammation would cause viral reactivation. These results suggest that the full active replication of the virus was not required for the observed increase in lung metastasis. Furthermore, mice actively infected with mCMV prior to tumor development also showed no positive detection of β-galactosidase activity in the tumors, and this indicates that breast cancer cell inoculation and tumor growth did not maintain mCMV infection at an active status. However, the immune response to the active infection at the time the tumor is developing could establish a microenvironment that reduces the metastatic phenotype, conferring some protection similar to heterologous immunity where infection with one pathogen can protect against another [12,13]. However, mRNA and β-galactosidase analyses were only performed at the experimental endpoint and in a limited number of tissues. It is possible that viral reactivation could have occurred once or multiple times prior to the endpoint and/or in other tissues where reactivation would have been quickly controlled by an intact memory immune response. Short phases of viral reactivation would have an impact on the immune responses that could play a role in metastasis. This explanation is likely since there is evidence of an active HCMV infection with HCMV proteins found in the metastatic sentinel lymph nodules of breast cancer patients [24]. Alternatively, proteins produced at a low level from latency associated transcripts, which include IE-1 in the absence of the transactivator IE-3 [36], or a splice variant of cmvIL-10 could also play a role in metastasis by controlling the immune response. It is also possible that the level of transcription for CMV mRNA was below the limit of detection with our current technique.

The effects of an mCMV infection on breast tumor progression were studied in BALB/c mice and C57BL/6 mice where we orthotopically injected syngeneic 4T1 or E0771 breast cancer cells, respectively, into mammary fat pads. C57BL/6 mice are less affected by infection with mCMV because of an effective natural killer cell response [37]. By contrast, BALB/c mice are more negatively affected by infection with mCMV because they lack the natural killer cell Ly49H activating receptor that recognizes an MHC class I-like glycoprotein produced during an mCMV infection [37]. This leads to an initial inefficient clearance of mCMV that impacts viral load and helps explain why we detected mCMV DNA more frequently in BALB/c than C57Bl/6 mice. Despite this greater sensitivity of BALB/c mice to mCMV infection, we only found an effect on metastasis in latently infected mice. The absence of an increased metastases in the mice acutely infected prior to tumor growth could have resulted from a more robust immune response to the virus when the syngeneic cancer cells were injected. If so, this only affected metastasis and not tumor growth. 

We also examined the effect of a latent mCMV infection in a mouse model with the spontaneous growth of multiple breast tumors. MMTV-PyVT mice express the polyomavirus middle T (PyVT) oncogene under the direction of the mouse mammary tumor virus (MMTV) promoter/enhancer [38]. These mice provided a very relevant model for human breast cancer since they could be infected early in life and the effects of the mCMV infection on the development of spontaneous breast tumors and metastasis could be determined.

We evaluated the effects of active, intermediate and latent infections with mCMV on breast tumor growth and metastasis in BALB/c and C57BL/6 models. The results in the BALB/c model were consistent in that there was mostly no significant effect of the mCMV infection regardless of timing on breast tumor volume or mass when compared to the control treatments. The significantly higher tumor mass in latently infected BALB/c mice compared to control mice treated with PBS is probably not important since there was no significant difference between the infected mice and those treated with a UV-inactivated virus (Figure 1A). Treatment with a UV-inactivated virus involved the injection of intact virus particles, which cannot replicate but can be recognized by the host immune system, providing a highly relevant control. In the C57BL/6 model, while there was no effect of intermediate or latent infection on tumor volume or mass, mCMV infection reduced tumor volume but not mass during an active infection. This is likely because the tumors in this model were less solid, reducing the accuracy of measurement with calipers.

The BALB/c model was established with an injection of 4T1 breast cancer cells, mimicking stage IV human breast cancer that form macro-metastases in the lungs [39]. The C57BL/6 model [40] and the MMTV-PyVT mice [41] develop much smaller metastases in the lungs and liver at a late stage of breast tumor development. As expected, our study in the C57BL/6 model showed no metastases in lungs and liver because of the ethical requirements to terminate our experiments at an earlier endpoint because of tumor size. The BALB/c and the MMTV-PyVT mice showed metastases in the lungs, which were increased significantly when the mice were latently infected with mCMV prior to tumor development. The increased metastases observed with a latent mCMV infection were not observed in the active or intermediate mCMV infection models. This work provides evidence from a mouse model that supports the contribution of HCMV infection to breast cancer metastasis and adds to the finding of a high rate of detection of HCMV proteins in the metastatic sentinel lymph nodules of breast cancer patients [24]. The establishment of these relevant preclinical models will also allow in-depth investigations into mechanisms. 

The results from the MMTV-PyVT mice confirmed that a latent mCMV infection had no significant effects on breast tumor growth and final tumor mass, but it did lead to worse phenotypes with the potential to promote metastasis. Multiple breast tumors developed in each MMTV-PyVT mouse, and we used the largest tumor for the endpoint measurements since these had almost identical sizes. There was a greater incidence of small fused breast tumors that were bloodier in the mCMV-infected mice, which could be indicative of a more severe phenotype. The breast tumors from mCMV-infected mice also had increased numbers of blood vessels, which is compatible with the overall increased bloodiness. Together, these results showed that a latent mCMV infection of MMTV-PyVT mice increased angiogenesis in breast tumors. This is a known feature associated with CMV infection [42,43], and it is important for metastasis [44]. Also, there were lower collagen levels in breast tumors from mCMV-infected mice, which is compatible with increased cancer cell egress [45]. In contrast, breast tumors from the actively or latently infected mCMV BALB/c model did not show these phenotype changes, including microvasculature and collagen content. This was possibly due to the differences between the type of breast tumor developed by using 4T1 cells, which results in a relatively solid tumor with a low blood content.

Cell proliferation, as measured by Ki67, was not significantly different in the breast tumors between the mCMV-infected and control-treated MMTV-PyVT or BALB/c mice. This result was compatible with our finding that the average size of the tumors was not significantly different. This was despite the greater vascularization of the breast tumors from MMTV-PyVT mice that presumably increased O_2_ and nutrient supply. By contrast, there was a greater cell proliferation in the lung micro-metastatic nodules of the infected MMTV-PyVT mice, which corresponds to the larger size of the nodules. The increased cell proliferation of lung nodules was only observed in the latently but not in the actively infected BALB/c model. These results were compatible with the increased numbers of macro-metastatic nodules that were seen only in the latently infected mice. The explanation for the specific effects of latent CMV infection prior to tumor growth in increasing metastasis could be in the specific expression of latency expressed viral proteins, and this will be the subject of future investigations. 

The FCMV control would have included cytokines and growth factors produced by the CMV-infected fibroblasts during CMV propagation. This control could also have contained small CMV viral fragments and small particles such as exosomes that were not removed by filtration. More importantly, the UV-inactivated control contained intact viral particles that were no longer infective. Both controls have the potential to stimulate a host immune response, which could have altered the tumor progression. However, similar effects were observed irrespective of using FCMV, UV inactivation or PBS controls.

The mechanisms for increased metastasis in mCMV-infected mice are probably complex, involving changes in the capacity of breast cancer cells to migrate from the primary tumor and to increase their ability to travel, establish and divide in distant sites. Along with changes in the tumor morphology that promote metastasis, mCMV-induced changes could also modify the host immune response so that cancer cells escape from immune destruction and aggravate tumor-promoting inflammation that favors tumor metastasis [46]. Our observation of the decreased IL-1α expression in the breast tumors of mCMV-infected MMTV-PyVT mice suggests that mCMV plays a role in immune regulation and affects the inflammatory condition at the breast tumor site. IL-1α also stimulates collagen synthesis [47,48], and lower concentrations of IL-1α in the tumors are compatible with the decreased collagen content. We also checked for evidence of an increased collagen breakdown, but there were no significant differences in the concentrations of the collagenase, MMP-8, in the tumors or for other MMPs (MMP-2 MMP-3, pro-MMP9 and MMPP-12) that could favor metastasis. 

Plasma concentrations of the pro-inflammatory cytokine, IL-6, were increased in mCMV-infected mice. This is a common feature associated with CMV infection [49] and is reported to have a role for carcinogenesis in salivary gland cancer [50]. An increase was also observed for IL-13, which promotes cell migration and invasion in colorectal cancer [51]. We observed a significant positive correlation between IL-6 and IL-13 in CMV-infected mice, and the significance of these changes are areas for future investigations. However, the present results provide important insights into potential mechanisms for HCMV infection and human breast cancer and metastasis. 

This study provides the first direct evidence from three mouse models of breast cancer that mCMV infection increases metastasis but does not affect the growth of primary tumors. This is compatible with the general conclusion that CMV is a tumor modulatory virus and not tumorigenic. Thus, the major finding was that a latent but not active mCMV infection prior to tumor development increases metastasis both in terms of the number of lung nodules and their size. This indicates that latent HCMV infections could play a crucial role in increasing mortality in breast cancer patients. Significantly, HCMV infects 40–70% of the adult population, but these numbers are reported to be as high as 95% in women with breast cancer. Little attention is given to the effects that HCMV infection could have on the current treatments of breast cancer. Our results provide evidence from mouse breast cancer models that latent mCMV infections prior to tumor development increase metastasis to the lungs. This work raises the possibility that treating the effects of HCMV infections or, alternatively, preventing HCMV infection altogether could substantially decrease metastasis and increase the life expectancy of breast cancer patients. 

## 4. Materials and Methods

### 4.1. Mouse Breast Cancer Models and Genotyping

Female BALB/c and C57BL/6 mice at 8 to 10 weeks of age were purchased from Charles River (Kingston, ON, Canada). Syngeneic orthotopic mouse breast cancer models were established as described [52], with the injection of 20,000 mouse 4T1 breast cancer cells (American Type Culture Collection, Manassas, VA, USA) in BALB/c mice and 10^6^ mouse E0771 breast cancer cells (CH3 BioSystems, Amherst, NY, USA) in C57BL/6 mice. A spontaneous mouse breast cancer model was generated by breeding male mice that were hemizygous for B6.FVB-Tg (MMTV-PyVT)634Mul/LellJ [38] with wild type female C57BL/6 mice. Ear notch samples were collected from weaned female mice, and the DNA was extracted using a DNeasy Blood & Tissue Kit (Qiagen, Toronto, ON, Canada). PCR was performed using the following program: initial denaturation for 30 sec at 98 °C, 35 cycles of denaturation for 10 sec at 98 °C, annealing for 20 sec at 64 °C, elongation for 25 sec at 72 °C and then a final elongation for 5 min at 72 °C. The reaction mixture contained 1 µL of DNA extract in a 25 μL reaction volume containing 2× PCR Taq Master Mix (Applied Biological Materials, Richmond, BC, Canada). Two sets of primers were added simultaneously to the reaction. Internal control: sense 5′-CAA ATG TTG CTT GTC TGG TG-3′ and antisense 5′-GTC AGT CGA GTG CAC AGT TT-3′ (gives 200 bp product). PyVT: sense 5′-GGA AGC AAG TAC TTC ACA AGG G-3′ and antisense 5′-GGA AAG TCA CTA GGA GCA GGG-3′ (gives 556 bp product). The primers were purchased from Integrated DNA Technologies (Coralville, IA, USA). The products were visualized after electrophoresis on a 2.0% agarose gel that was stained with ethidium bromide. Female mice that were positive for both the internal control and PyVT were used for the experiments.

The mice were housed in Animal Biosafety Level 2 containment in the Health Sciences Laboratory Animal Services facility at the University of Alberta. All procedures were approved by the University of Alberta Animal Welfare Committee and followed guidelines from the Canadian Council of Animal Care.

### 4.2. Preparation of mCMV

RM427+, a strain of mCMV containing an insertion of the LacZ gene in the nonessential immediate-early 2 gene, was used for infection (gift from E. Mocarski, Stanford University, Stanford, CA, USA) [27]. The infection with RM427+ did not differ from the native virus strain [27]. The virus was propagated in mouse fibroblasts and stored as aliquoted viral stocks at −80 °C as previously described [27]. A filtered control (FCMV) was generated by passing the propagated mCMV through a 0.05 μm membrane filter (Millipore Sigma, Tullagreen, Carrigtwohill Co. Cork, IRL) to eliminate the viral particles but to retain the propagation medium. A UV-inactivated control was generated by irradiating the propagated mCMV with a 230-volt UV lamp from 20 cm for 30 min. Both control solutions showed no viral infection when cultured with mouse fibroblasts.

### 4.3. Mouse Infection with mCMV

mCMV (10^6^ plaque-forming units, PFU) was injected i.p. in 100 μL [27] in mice chosen at random. An equal volume of phosphate-buffered saline (PBS), FCMV or the UV-inactivated virus was injected as a control into the other mice. 

Each strain of mouse was tested for a successful active infection using separate test animals injected with the controls described above or mCMV. The mice were then euthanized after 4 days, and the kidneys were harvested, embedded in an optimal cutting temperature compound (Fisher Scientific International Inc., Ottawa, ON, Canada) and stored at −80 °C. The LacZ gene inserted into the IE-2 gene of mCMV produced β-galactosidase when the virus was actively replicating, and this was visualized by its activity in converting X-gal into a blue product. The kidneys were sectioned and stained for β-galactosidase activity using the LacZ Detection Kit for Tissues (InvivoGen, San Diego, CA, USA) following the manufacturer’s instructions. Latency was defined as the presence of mCMV genome but the absence of mRNA for gB, a transcript produced in the late phase of infection but not produced in latency [53].

The infection of BALB/c and C57BL/6 mice was for 4 days, 11 days or 10 weeks to generate an active, intermediate or latent infection, respectively, before injecting E0771 or 4T1 breast cancer cells, respectively into a mammary fat pad [52]. The MMTV-PyVT mice were infected at 5 weeks of age, allowing at least 10 weeks for a latent infection to develop before spontaneous breast tumors were first palpable.

### 4.4. Tumor Growth and Tissue Collection

Tumor growth was monitored by taking orthogonal caliper measurements in two dimensions and estimating the tumor volume by width^2^ × length/2. The syngeneic-orthotopic models had a single tumor develop per mouse, and the experimental endpoint was 25 days post-cancer cell inoculation so the estimated tumor volume was approximately 1000 mm^3^. The MMTV-PyVT mice developed multiple spontaneous tumors with a high intrinsic variability, and the experimental endpoint was when the largest tumor reached 4000 mm^3^. Salivary glands and spleens were immediately snap frozen in liquid nitrogen and stored at −80 °C. The tumors were isolated and weighed, and their morphological characteristics assessed by two independent observers who were blinded to the treatment group. The amount of blood in the tumor was categorized as pale, moderate or bloody, with the tumors being pale, pink or dark red in color, respectively. The number of lobes on the tumor was categorized as single tumor, multilobed (lobes started to form on the original tumor) or fused small tumors (fusion of largely grown lobes). Further characterization of each tumor was made with respect to morphology including whether they were solid, soft, necrotic, disintegrated or fluid-filled. Half of each tumor was snap frozen, and the other half was fixed in 10% formalin. 

### 4.5. Metastasis

The lungs from BALB/c mice were stained with India ink (American MasterTech, Lodi, CA, USA) by intratracheal injection, leaving the metastatic nodules as white against the black lung [54]. The total number of nodules observed on the lung surface was counted. The nodules were then carefully isolated from the lung tissue under a dissection microscope for analysis to detect mCMV. The livers and lungs from C57BL/6 and MMTV-PyVT mice were fixed with 10% formalin. Lung micro-metastasis was determined using fixed lung specimens that were paraffin-embedded. Sample slices were obtained from the front, middle and back sections of the lung and stained with hematoxylin and eosin to identify tumor nodules. Briefly, hydrated slides were stained with hematoxylin for 3 min, rinsed with running tap water for 5 min, differentiated with 0.3% concentrated hydrochloric acid in 80% ethanol, stained with eosin for 45 sec and dehydrated. The images were taken from 5 fields per section with a 5× magnification lens, and staining was quantified by ImageJ (National Institutes of Health, Bethesda, MD, USA) for the number of nodules per microscopic field and area of nodules relative to the corresponding lung area. Liver micro-metastasis was examined using the same technique.

### 4.6. Immunohistochemistry

Breast tumors and lungs fixed in 10% formalin were embedded in paraffin and sectioned. The hydrated slides were microwaved in a Tender Cooker (Nordic Ware, Minneapolis, MN, USA) for 30 min in 10 mM citric acid (pH 6.0) for antigen retrieval. A Rabbit-specific HRP/DAB (ABC) Detection IHC Kit (Abcam, Toronto, ON, Canada) was used according to the manufacturer’s instructions. The rabbit anti-CD31 (ab28364, 1:80) antibody was from Abcam (Toronto, ON, Canada), and the rabbit anti-Ki67 (D3B5, 1:200) was from Cell Signaling Technology (Whitby, ON, Canada). Positive staining events for CD31 and Ki67 were selected and counted using ImageJ. An analysis was made using images taken with a 5× magnification lens, and the illustration was taken with a 20× magnification lens. CD31 was quantified as the number of vessels per field, and Ki67 as a percentage of the positive cells. The results were averaged from 10 image fields for each sample.

### 4.7. Picro-Sirius Red Staining for Collagen

Sectioned breast tumor slides were hydrated and incubated for 1 h with 0.1% Sirius Red F3B (Sigma-Aldrich, Oakville, ON, Canada) dissolved in a saturated aqueous solution of picric acid, washed twice with 0.5% acetic acid and then dehydrated [55]. Collagen type I, III and IV were stained red against a pale orange background when visualized under light microscope. Areas of positive staining were quantified using Adobe Photoshop (Adobe Inc., San Jose, CA, USA) and Image J software. The results were averaged from 10 image fields for each sample.

### 4.8. mCMV Viral DNA Detection

The tissues were isolated for DNA analysis using the DNeasy Blood & Tissue Kit (Qiagen, Toronto, ON, Canada). The presence of mCMV DNA was detected using primer sets from Integrated DNA Technologies (Coralville, IA, USA) that were specific for the late gene mCMV gB [56]. The mCMV gB external primers were sense 5′-TCA TCA ACT CGA CGA AGC TC-3′ and antisense 5′-ATC TCG TCC AGG CTG AAC AC-3′ (giving a 298-bp product), and the mCMV gB internal primers were sense 5′-CTG GGC GAG AAC AAC GAG AT-3′ and antisense 5′-CGC AGC TCT CCC TTC GAG TA-3′ (giving a 235-bp product). The detection of mCMV DNA from the IE-1 region was also performed, the external primers were sense 5′-TGG ATG AGA ACC GTG TCT AC-3′ and antisense 5′-ATA TCA TCT TGC GTT GTC TT-3′ (giving a 549-bp product) and the internal primers were sense 5′-TAC AGG ACA ACA GAA CGC TC-3′ and antisense 5′-CCT CGA GTC TGG AAC CGA AA-3′ (giving a 310-bp product) [57]. Nested PCR was performed for both sets of primers using the 2× PCR Taq Master Mix (Applied Biological Materials, Richmond, BC, Canada) in a 25 μL reaction volume. The extracted DNA (300 ng) was amplified with the external primer set for the first round of amplification and then 2 μL of the amplified product was used with the internal primer set for the second round of amplification. Both rounds of PCR were performed with the following program: initial denaturation for 4 min at 94 °C, 35 cycles of denaturation for 30 sec at 94 °C, annealing for 30 sec at 53 °C for gB and 60 °C for IE-1, elongation for 30 sec at 72 °C and then a final elongation for 7 min at 72 °C. The internal control used was β-actin (gives 150-bp product): sense 5′-ATT GTG ATG GAC TCC GGT GA-3′ and antisense 5′-AGC TCA TAG CTC TTC TCC AG-3′. The products were detected after electrophoresis on a 1.5% agarose gel that was stained with ethidium bromide. 

### 4.9. mCMV mRNA Detection

The tissues were homogenized in 1 mL of Trizol (Invitrogen Life Technologies, Grand Island, NY, USA) contained in 2 mL microcentrifuge tubes with 5-mm stainless steel beads, using the Qiagen TissueLyser II system (24-sample plates, 25 Hz, 5 min) (Qiagen, Toronto, ON, Canada). The RNA was isolated using the ultRNA Column Purification kit (Applied Biological Materials, Richmond, BC, Canada). cDNA was produced by PCR using Reverse Transcription master mix, and the relative amount of the target gene was quantified by Real-Time PCR using EvaGreen qPCR master mix (Applied Biological Materials, Richmond, BC, Canada). The relative abundance of mCMV gB expression at the mRNA level was determined by normalizing against the housekeeping gene, glyceraldehyde 3-phosphate dehydrogenase (GAPDH). mCMV gB (primer for mRNA): sense 5′-AGG GCT TGG AGA GGA CCT ACA-3′ and antisense 5′-GCC CGT CGG CAG TCT AGT C-3′. mCMV IE-1/3: sense 5′-GTA CAA AAG GTC AAT AGG GG-3′ and antisense 5′-GTA CCG ACG CTG GTC GCG CC-3′. GAPDH: sense 5′-TCC TGC ACC ACC AAC TGC TT-3′ and antisense 5′-TCT TAC TCC TTG GAG GCC AT-3′. 

### 4.10. Cytokine/Chemokine and Matrix Metalloproteinase (MMP) Expressions

The breast tumors (20 mg) were homogenized in 200 µL of radioimmunoprecipitation buffer (50 mM Tris-HCl (pH 7.4), 1% NP-40, 0.25% Na-deoxycholate, 150 mM NaCl, 1 mM EDTA, 1:100 dilution of protease inhibitor) and centrifuged for 20 min at 4 °C. The supernatants were then transferred to a fresh tube. Protein concentration was measured using the bicinchoninic acid protein assay (Thermo Fisher Scientific, Rockford, IL, USA) and adjusted to 1 mg/mL. This protein solution and plasma samples were analyzed using a Mouse Cytokine/Chemokine Discovery array 32-plex and for MMP expression by Eve Technologies Corp (Calgary, AB, Canada).

### 4.11. Statistics

Results other than tumor characteristics in the MMTV-PyVT model are expressed as means ± SEM. Student’s t-test, one-way ANOVA or two-way ANOVA were used as appropriate and indicated in each figure legend. *p* < 0.05 was considered significant as evaluated and graphed using GraphPad Prism 6 (GraphPad Software, La Jolla, CA, USA). Changes in the number of tumors by blood content, type of tumors and morphology (severity characteristics) across infection status (with/without mCMV infection) were estimated by a Chi-square analysis (and Fisher’s exact test when applicable). An additional logistic model was used to assess the odds ratio of severity characteristics between the with/without mCMV infection groups as suggested by the results of the Chi-square analysis. The Chi-square analysis and the logistic models were performed using SPSS version 25.0 [58] and *p*-values of <0.05 were considered to be statistically significant.

## 5. Conclusions

This study provides the first direct evidence from mouse breast cancer models that latent mCMV infections prior to tumor development increase metastasis to the lungs but do not affect tumor growth. This work raises the possibility that treating the effects of HCMV infections or, alternatively, preventing HCMV infection altogether could substantially decrease metastasis and increase the life expectancy of breast cancer patients. 

## Figures and Tables

**Table 1:** The average tumor volume, mass and density in MMTV-PyVT mice with and without a latent mCMV infection.

Variables	n	Mean ± SD	95% CI	*p*-Value
Tumor Volume (mm^3^)				
FCMV	133	957 ± 947	795, 1120	0.064
CMV	104	1202 ± 1077	993, 1411	
Tumor Mass (mg)				
FCMV	133	0.84 ± 0.85	0.70, 0.99	0.164
CMV	104	1.01 ± 0.96	0.82, 1.20	
Tumor Density				
FCMV	133	0.90 ± 0.29	0.85, 0.95	0.789
CMV	104	0.89 ± 0.33	0.82, 0.95	

## Supplementary Materials

The following are available online at https://www.mdpi.com/2072-6694/11/4/447/s1, Table S1: Severity of tumors in MMTV-PyVT mice with and without latent mCMV infection, Figure S1: Verification of successful mCMV infection, Figure S2: Estimated volume of breast tumors developed over time in BALB/c and C57BL/6 mice with and without active, intermediate or latent mCMV infections, Figure S3: Breast tumor mass and lung metastasis were not significantly affected in mice with active or intermediate mCMV infections, Figure S4: Evidence of enhanced cancer cell proliferation in lung metastatic nodules of BALB/c mice latently infected prior to cancer cell injection, but not in the mice actively infected prior to cancer cell injection, Table S2: Detection of mCMV glycoprotein B DNA in specific tissues, nodules or tumors from each mouse in all animal groups.

## Abbreviations

CMV	cytomegalovirus
FCMV	filtered cytomegalovirus
GAPDH	glyceraldehyde 3-phosphate dehydrogenase
gB	glycoprotein B
HCMV	human cytomegalovirus
IE	immediate early
IL	interleukin
mCMV	mouse cytomegalovirus
MMP	matrix metalloproteinase
MMTV	mouse mammary tumor virus
PyVT	polyomavirus middle T
