# Supplementary material for: Latent Cytomegalovirus Infection in Female Mice Increases Breast Cancer Metastasis
Source: Cancers (Basel). 2019 Mar 29;11(4):447. doi: 10.3390/cancers11040447 (PMC6520675; doi:10.3390/cancers11040447)
Supplement: Supplementary file 1 [file cancers-11-00447-s001.zip › cancers-433708-supplementary.pptx]

## Slide 1
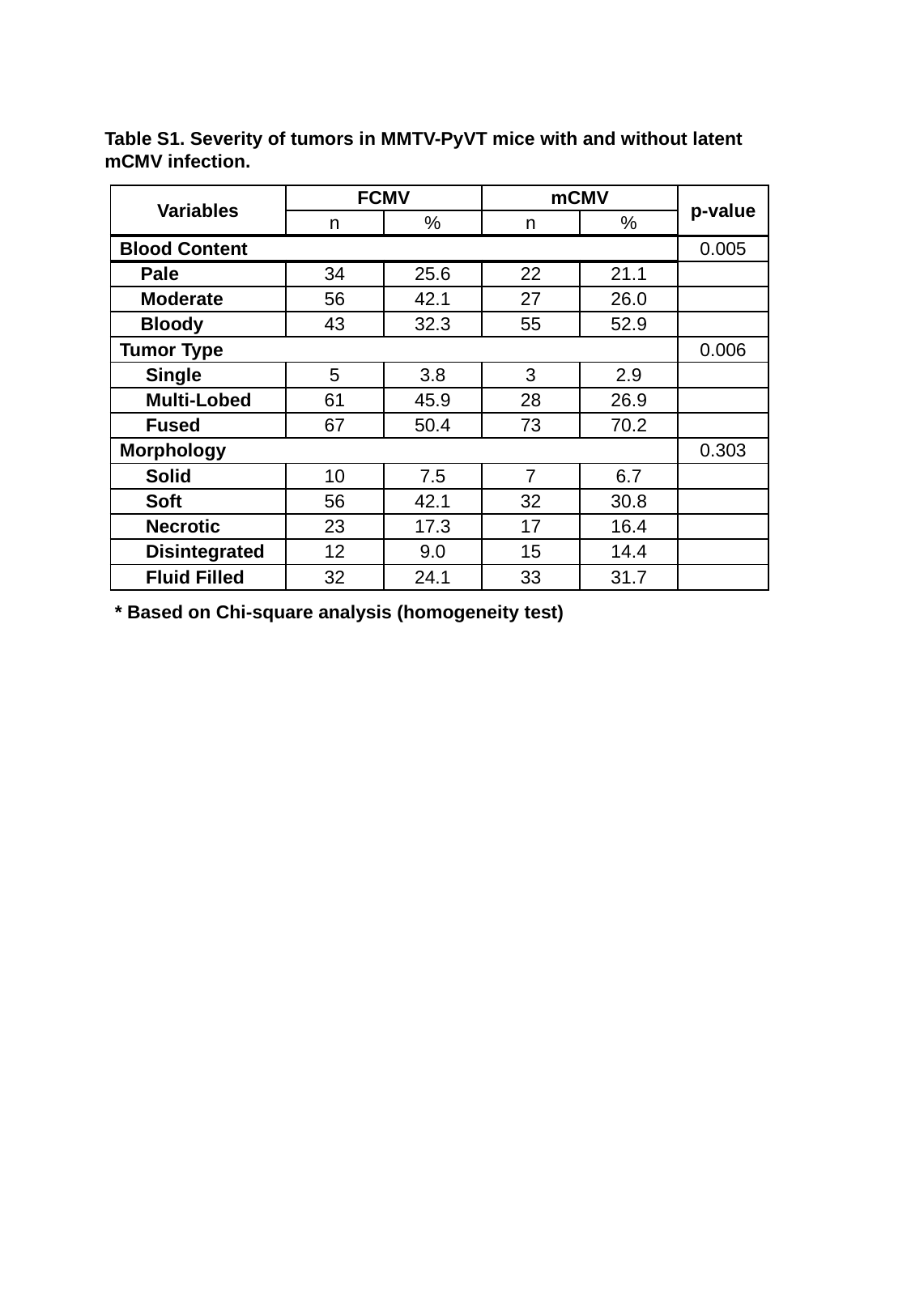

Table S1. Severity of tumors in MMTV-PyVT mice with and without latent mCMV infection.
| Variables | FCMV | | mCMV | | p-value |
| --- | --- | --- | --- | --- | --- |
| | n | % | n | % | |
| Blood Content | | | | | 0.005 |
| Pale | 34 | 25.6 | 22 | 21.1 | |
| Moderate | 56 | 42.1 | 27 | 26.0 | |
| Bloody | 43 | 32.3 | 55 | 52.9 | |
| Tumor Type | | | | | 0.006 |
| Single | 5 | 3.8 | 3 | 2.9 | |
| Multi-Lobed | 61 | 45.9 | 28 | 26.9 | |
| Fused | 67 | 50.4 | 73 | 70.2 | |
| Morphology | | | | | 0.303 |
| Solid | 10 | 7.5 | 7 | 6.7 | |
| Soft | 56 | 42.1 | 32 | 30.8 | |
| Necrotic | 23 | 17.3 | 17 | 16.4 | |
| Disintegrated | 12 | 9.0 | 15 | 14.4 | |
| Fluid Filled | 32 | 24.1 | 33 | 31.7 | |
* Based on Chi-square analysis (homogeneity test)

## Slide 2
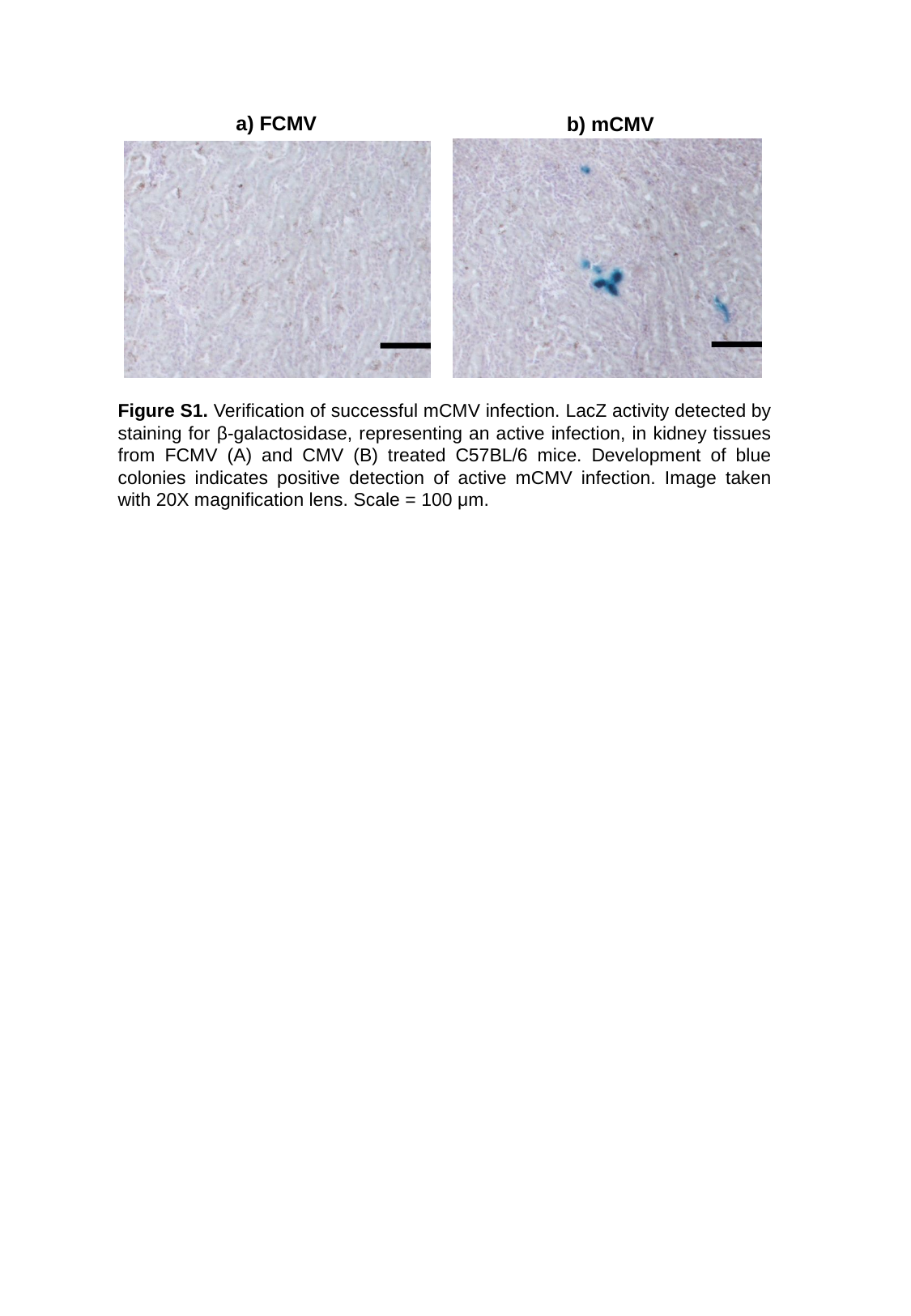

a) FCMV
b) mCMV
Figure S1. Verification of successful mCMV infection. LacZ activity detected by staining for β-galactosidase, representing an active infection, in kidney tissues from FCMV (A) and CMV (B) treated C57BL/6 mice. Development of blue colonies indicates positive detection of active mCMV infection. Image taken with 20X magnification lens. Scale = 100 μm.

## Slide 3
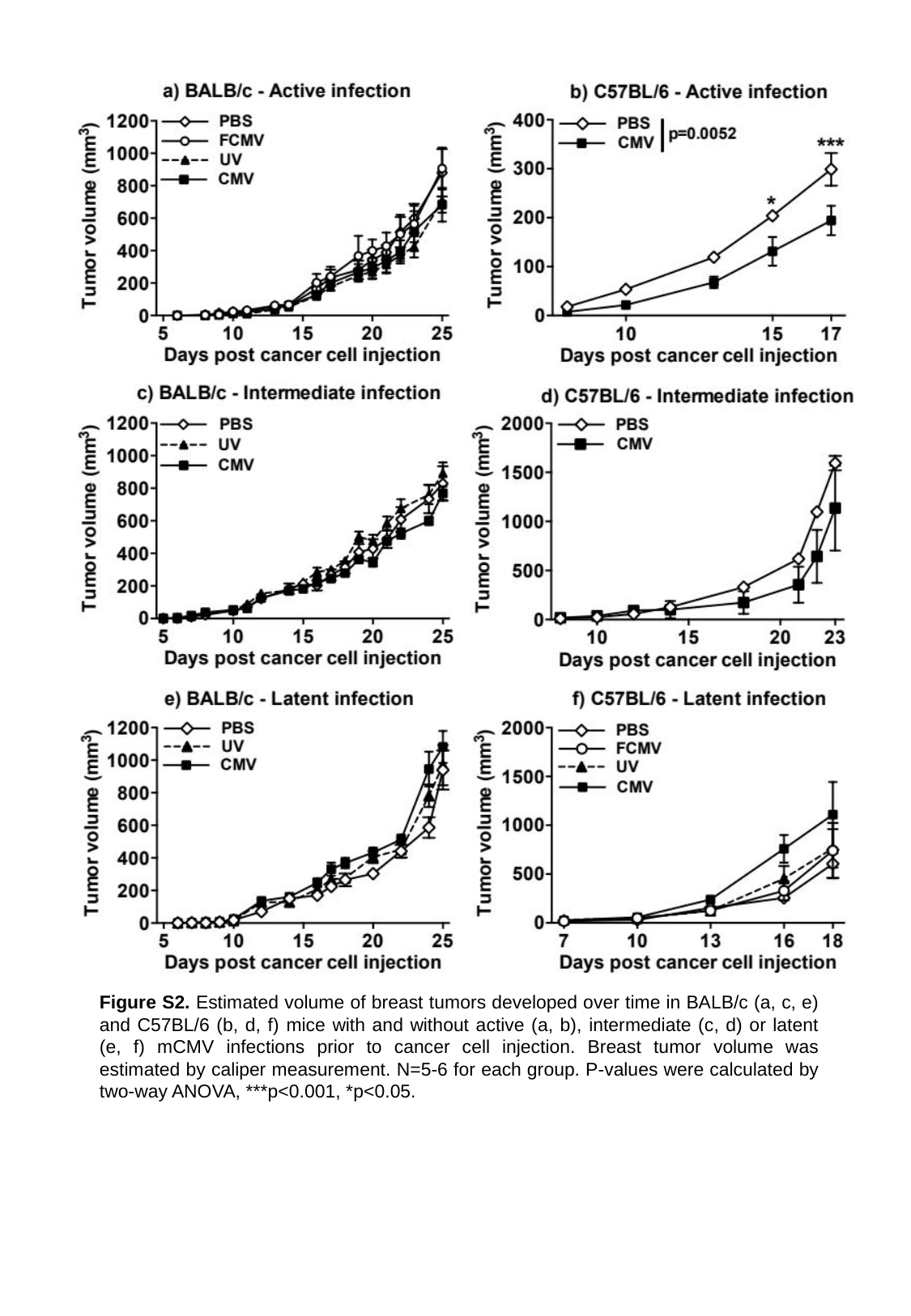

Figure S2. Estimated volume of breast tumors developed over time in BALB/c (a, c, e) and C57BL/6 (b, d, f) mice with and without active (a, b), intermediate (c, d) or latent (e, f) mCMV infections prior to cancer cell injection. Breast tumor volume was estimated by caliper measurement. N=5-6 for each group. P-values were calculated by two-way ANOVA, ***p<0.001, *p<0.05.

## Slide 4
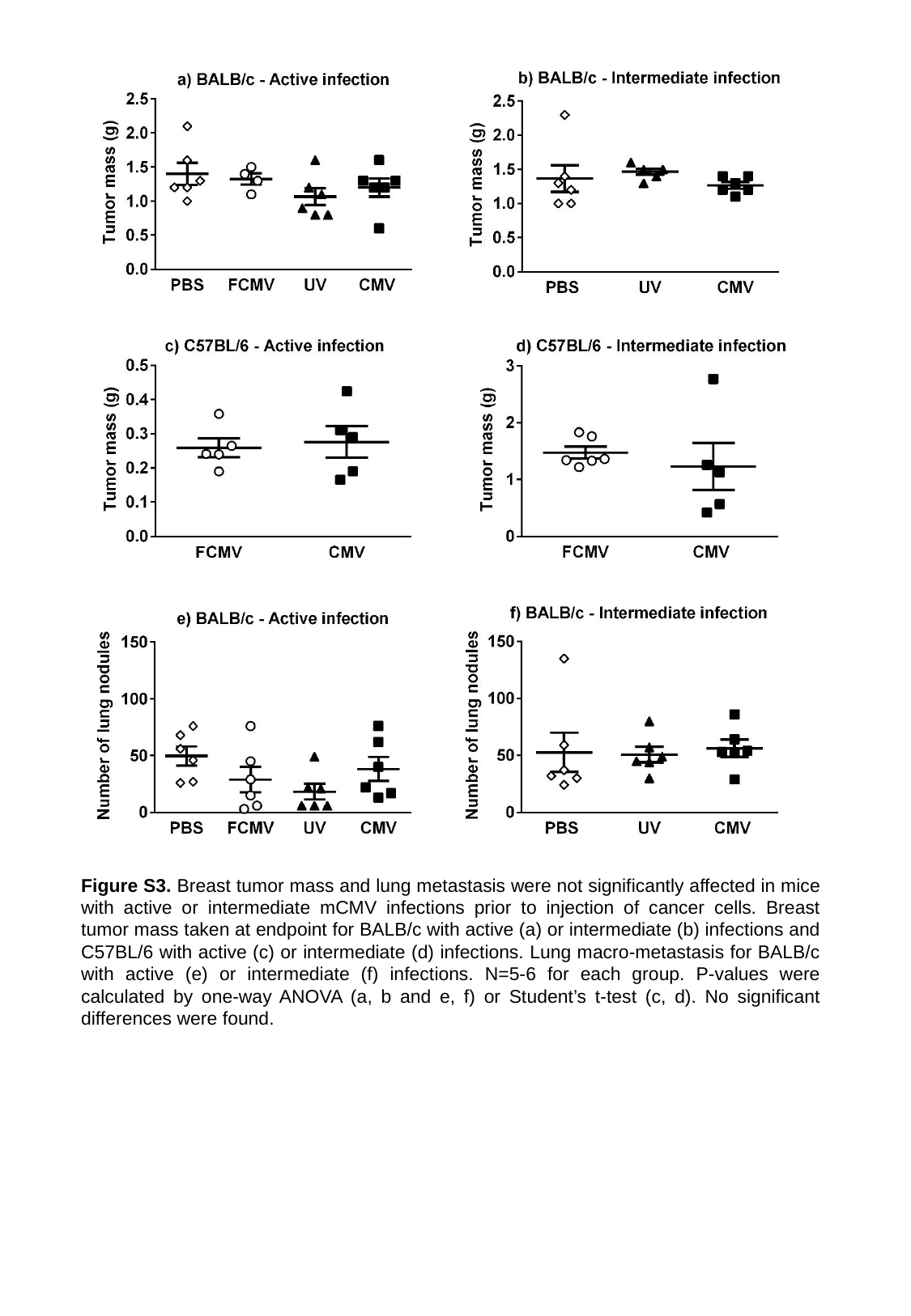

Figure S3. Breast tumor mass and lung metastasis were not significantly affected in mice with active or intermediate mCMV infections prior to injection of cancer cells. Breast tumor mass taken at endpoint for BALB/c with active (a) or intermediate (b) infections and C57BL/6 with active (c) or intermediate (d) infections. Lung macro-metastasis for BALB/c with active (e) or intermediate (f) infections. N=5-6 for each group. P-values were calculated by one-way ANOVA (a, b and e, f) or Student’s t-test (c, d). No significant differences were found.

## Slide 5
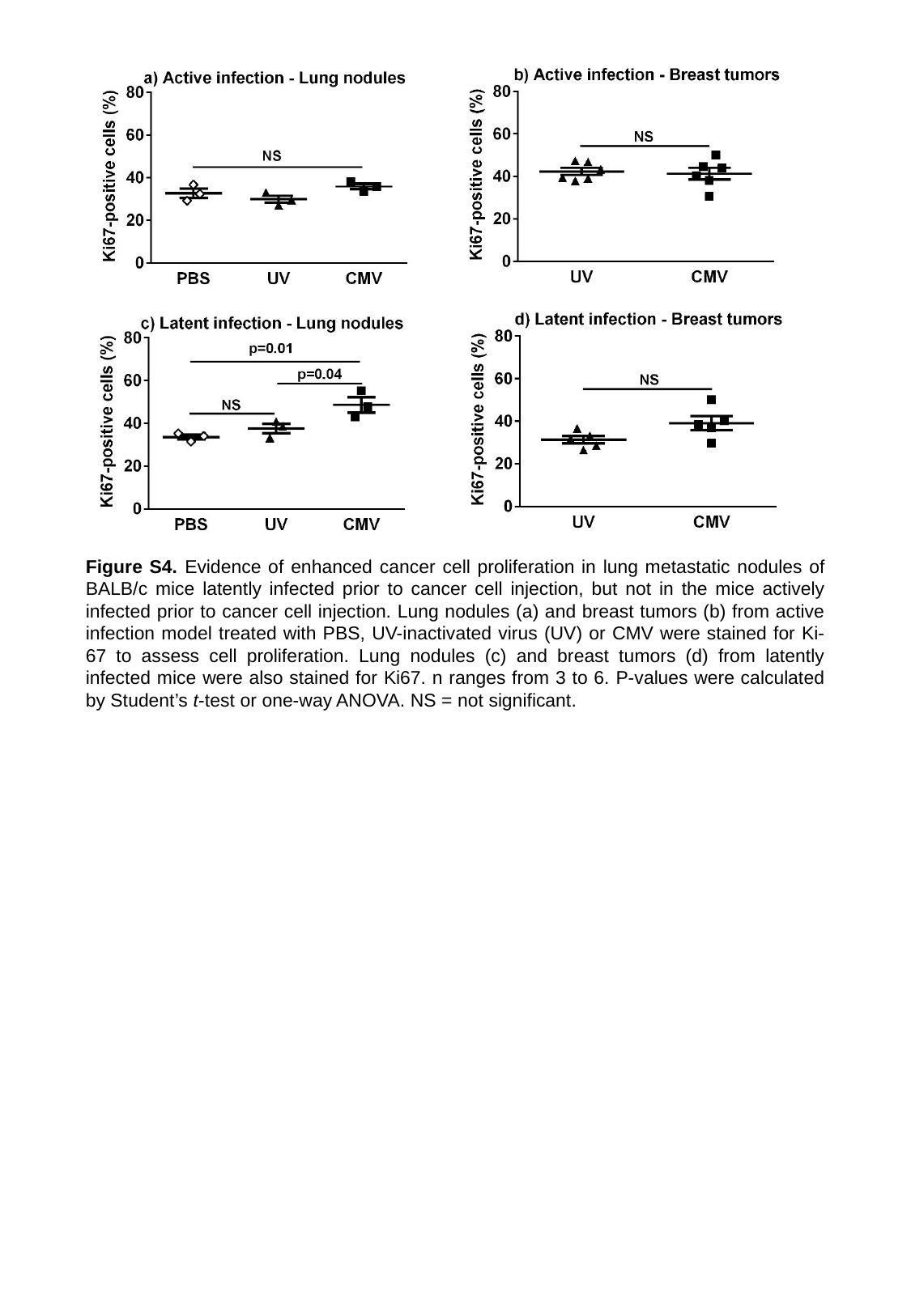

Figure S4. Evidence of enhanced cancer cell proliferation in lung metastatic nodules of BALB/c mice latently infected prior to cancer cell injection, but not in the mice actively infected prior to cancer cell injection. Lung nodules (a) and breast tumors (b) from active infection model treated with PBS, UV-inactivated virus (UV) or CMV were stained for Ki-67 to assess cell proliferation. Lung nodules (c) and breast tumors (d) from latently infected mice were also stained for Ki67. n ranges from 3 to 6. P-values were calculated by Student’s t-test or one-way ANOVA. NS = not significant.

## Slide 6
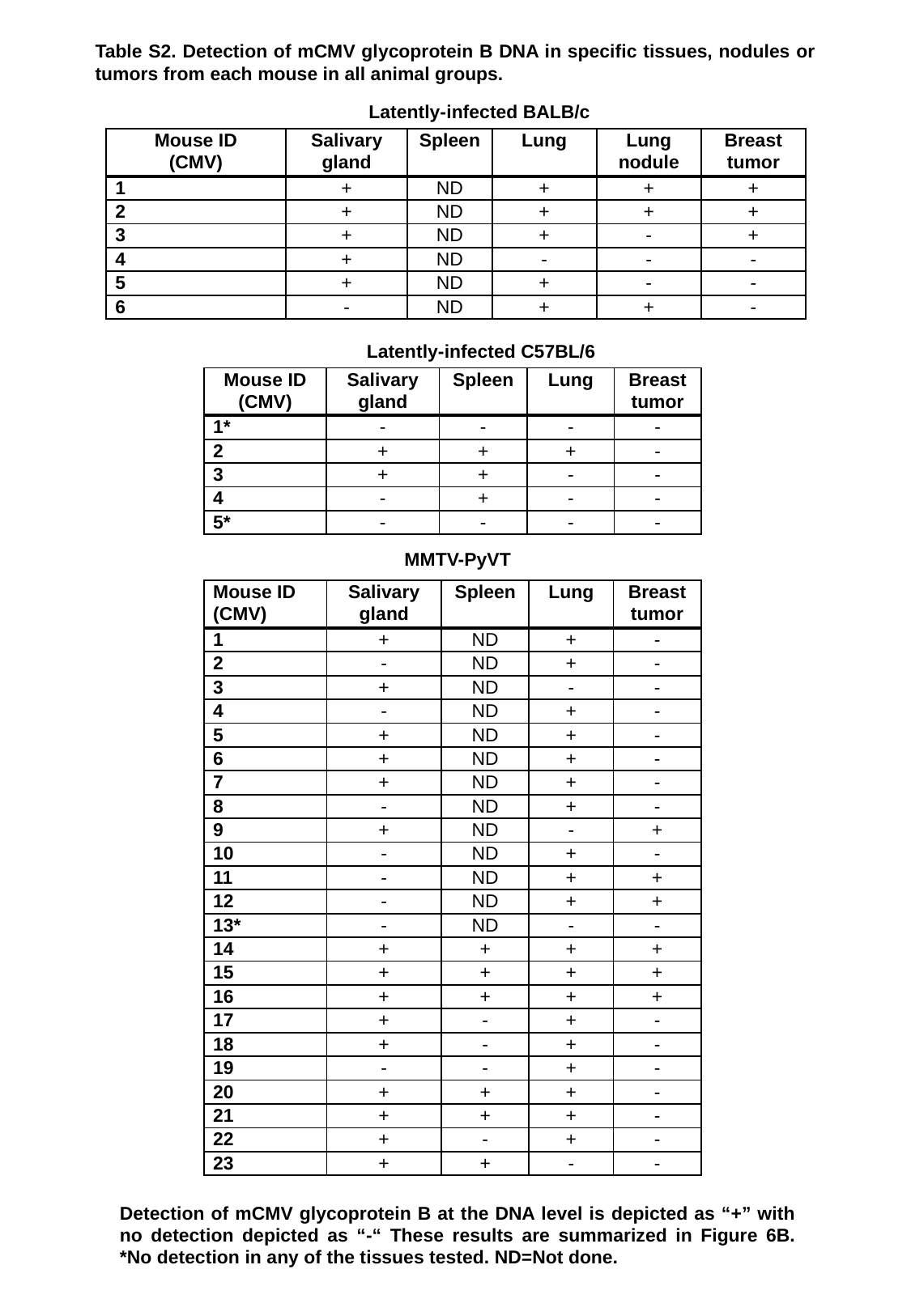

Table S2. Detection of mCMV glycoprotein B DNA in specific tissues, nodules or tumors from each mouse in all animal groups.
Latently-infected BALB/c
| Mouse ID (CMV) | Salivary gland | Spleen | Lung | Lung nodule | Breast tumor |
| --- | --- | --- | --- | --- | --- |
| 1 | + | ND | + | + | + |
| 2 | + | ND | + | + | + |
| 3 | + | ND | + | - | + |
| 4 | + | ND | - | - | - |
| 5 | + | ND | + | - | - |
| 6 | - | ND | + | + | - |
Latently-infected C57BL/6
| Mouse ID (CMV) | Salivary gland | Spleen | Lung | Breast tumor |
| --- | --- | --- | --- | --- |
| 1\* | - | - | - | - |
| 2 | + | + | + | - |
| 3 | + | + | - | - |
| 4 | - | + | - | - |
| 5\* | - | - | - | - |
MMTV-PyVT
| Mouse ID (CMV) | Salivary gland | Spleen | Lung | Breast tumor |
| --- | --- | --- | --- | --- |
| 1 | + | ND | + | - |
| 2 | - | ND | + | - |
| 3 | + | ND | - | - |
| 4 | - | ND | + | - |
| 5 | + | ND | + | - |
| 6 | + | ND | + | - |
| 7 | + | ND | + | - |
| 8 | - | ND | + | - |
| 9 | + | ND | - | + |
| 10 | - | ND | + | - |
| 11 | - | ND | + | + |
| 12 | - | ND | + | + |
| 13\* | - | ND | - | - |
| 14 | + | + | + | + |
| 15 | + | + | + | + |
| 16 | + | + | + | + |
| 17 | + | - | + | - |
| 18 | + | - | + | - |
| 19 | - | - | + | - |
| 20 | + | + | + | - |
| 21 | + | + | + | - |
| 22 | + | - | + | - |
| 23 | + | + | - | - |
Detection of mCMV glycoprotein B at the DNA level is depicted as “+” with no detection depicted as “-“ These results are summarized in Figure 6B. *No detection in any of the tissues tested. ND=Not done.
